# Impact of changes in leisure activities on incident disability among older adults: a nationwide cohort study

**DOI:** 10.7189/jogh.15.04289

**Published:** 2025-10-31

**Authors:** Chi Zhang, Jin Liu, Anying Bai, Yuting Kang, Qiang Gao, Jie Zhang, Yushan Zhang, Wenyu Li, Yingqi Zhao, Ping Zeng, Ji Shen

**Affiliations:** 1 Key Laboratory of Geriatrics, Beijing Institute of Geriatrics, Institute of Geriatric Medicine, Chinese Academy of Medical Sciences, Beijing Hospital, National Center of Gerontology of National Health Commission, Beijing, China; 2 School of Public Health, Sun Yatsen University, Guangzhou, China; 3 School of Population Medicine and Public Health, Chinese Academy of Medical Sciences and Peking Union Medical College, Beijing, China; 4 Department of Science Research, Beijing Hospital, National Center of Gerontology, Institute of Geriatric Medicine, Chinese Academy of Medical Sciences, Beijing, China; 5 Department of Geriatrics, Beijing Hospital, National Center of Gerontology, Institute of Geriatric Medicine, Chinese Academy of Medical Sciences, Beijing, China

## Abstract

**Background:**

As research has shown that participation in leisure activities (LAs) is closely related to health outcomes in later life, we explored the associations between dynamic changes in LAs and disability in activities of daily living (ADL) in older people.

**Methods:**

We enrolled 11627 older adults free of disability from six waves in the Chinese Longitudinal Healthy Longevity Survey (1998, 2000, 2002, 2005, 2008, and 2011). All participants completed two consecutive LAs measurements (including six typical activities) in the first two waves and were divided into four categories according to the patterns of change: sustained low LA (n = 2931), LA increase (n = 1729), LA decrease (n = 1802), and sustained high LA (n = 5169). Incident ADL disability was identified using a total Katz index <6 points during each follow-up until 2018. We used Cox proportional hazard regressions to test the association between changes in LAs and disability, with demographic characteristics, health behaviours, and chronic diseases included as covariates.

**Results:**

During 51991.85 person-years of follow-up, 3717 participants developed ADL disability. Compared with the sustained low group, the adjusted hazard ratio (aHR) of disability for the sustained high group, LA increase group, and LA decrease group were 0.62 (95% CI = 0.57–0.69), 0.66 (95% CI = 0.59–0.74), and 1.01 (95% CI = 0.91–1.11), respectively. Specifically, increased participation in outdoor activities, keeping domestic animals or pets, and playing cards or mahjong were factors associated with a lower risk of disability. The main results remained stable in the subgroup and sensitivity analyses.

**Conclusion:**

Maintaining high participation and increasing participation in LAs are associated with a lower risk of ADL disability in community-dwelling older individuals. Therefore, promoting participation in LAs represents a practical strategy to prevent ADL disability, thereby contributing to healthy ageing.

According to the World Health Organization, the global population aged ≥60 years is projected to reach 2.1 billion by 2050, with two-thirds living in low- and middle-income countries [1]. China, one of the world’s most populous countries, was home to nearly 297 million individuals from this age group in 2023, accounting for 21.1% of the total population [2]. The incidence of disability rises with population ageing, markedly increasing mortality risk among older individuals [3,4]. Older adults with disabilities are also more prone to depression and anxiety, while their care imposes substantial economic and psychological burdens on caregivers, potentially harming their quality of life and health [5,6].

Promoting participation in diverse community-based leisure activities (LAs) is a practical strategy to support healthy ageing and mitigate disability in activities of daily living (ADL). Evidence suggests that LAs are vital for preventing disability and reducing mortality in older adults [7,8]. For example, older individuals engaging in sufficient physical activity had a lower risk of disability over a 24-month follow-up [9]. A 20-year population-based cohort study in China found that healthy lifestyles, including regular physical exercise, were associated with lower mortality risk and longer life expectancy [2].

While previous cohort studies have highlighted the benefits of LAs, they have mainly relied on single-time point measurements, failing to capture longitudinal trajectories. A nationwide cohort study in China demonstrated that even among the oldest-old population, higher engagement in LAs was associated with lower risk of disability [10,11]. Evidence also remains sparse regarding the role of maintaining or increasing LA participation over time in reducing disability risk, which could be crucial given the dynamic nature of ageing and the constant changes in health status and living environments. Cultural considerations should also be integrated into studies on LAs and healthy ageing to generate contextually relevant evidence. Such findings could inform future research and interventions aimed at fostering sustainable practices and maximising LA engagement among older adults.

Considering these limitations, we used longitudinal data from the Chinese Longitudinal Healthy Longevity Survey (CLHLS) to analyse the association of changes in LAs with incident physical disability among a representative sample of community-dwelling older adults from China. We also aimed to explore the effect of specific types of LAs on the risk of incident disability.

## METHODS

### Study population

For this longitudinal analysis, we included all new participants from the six waves (1998, 2000, 2002, 2005, 2008, and 2011) of the CLHLS who had completed at least three follow-up surveys. Data on the change in LAs were collected specifically during the first two study waves using standardised scales. In the subsequent waves, we gathered information on the incidence of ADL disability. We defined the analytical sample by applying several exclusion criteria. At baseline, individuals were excluded if they: were younger than 60 years of age; had missing data on LAs in the first wave; or had a reported disability in ADL at the time of enrolment. Following this, participants were also excluded from the final analysis if they lacked LAs data in the first two waves or did not provide any ADL information during the subsequent follow-up period.

In the initial and the second waves, the frequency of participation in LAs was collected continuously twice using standardised scales, while ADL information was gathered in subsequent follow-ups. We first included 43583 individuals from the initial CLHLS dataset. Then, we excluded 130 participants who were <60 years old and 36 who were missing LA data in the first wave. In the second wave, 13 086 individuals died and 5946 were lost to follow-up, leaving 24385 observations. After excluding 46 individuals lacking LA data in the second wave and 2501 who had reported disabilities at baseline, 21838 participants remained in the follow-up. In the first follow-up wave, 7079 individuals died, 2816 were lost to follow-up and 316 failed to provide any ADL information. The Biomedical Ethics Committee of Peking University (IRB00001052-13074) approved the CLHLS, and all participants or guardians provided informed consent. We confirm that our reanalysis of the CLHLS dataset adheres to the Journal of Global Health’s Guidelines for Reporting Analyses of Big Data Repositories Open to the Public (Table S9 in the **Online Supplementary Document**).

### Measurement of LAs

Engagement in six typical LAs was assessed through face-to-face interviews, which included three physical activities (gardening, engaging in outdoor activities, and keeping domestic animals or pets) and three cognitive activities (reading newspapers or books, playing cards or mahjong, and watching TV or listening to the radio). The frequency of participation in each activity was categorised as ‘almost every day = 2’, ‘sometimes = 1’, and ‘never = 0’. The total LA score ranged from 0 to 12, with higher scores indicating more frequent participation overall. The LA scale was designed to be culturally relevant and user-friendly for Chinese older adults; here, it had a Cronbach’s alpha coefficient of 0.865, demonstrating high reliability. We divided the participants into low-activity (0–5 points) and high-activity (6–12 points) groups based on the median value of the total LA score. We also categorised them into four groups according to changes in the LA scores over two repeated measurements: sustained high (high to high), LA increase (low to high), LA decrease (high to low), and sustained low (low to low).

To analyse changes in each specific LA, we created four distinct groups: sustained non-participation (‘never’ to ‘never’), initiation (‘never’ to ‘sometimes/almost every day’), cessation (‘sometimes/almost every day’ to ‘never’), and sustained participation (‘sometimes/almost every day’ to ‘sometimes/almost every day’). The sustained non-participation group served as the reference category in these analyses.

### ADL disability

In each follow-up after the second wave, the CLHLS collected data on six activities of daily living (eating, dressing, bowel and bladder control, toileting, bathing, and transferring from bed to chair) using the Katz Index. Independence in completing an activity was scored as one point, while dependence or partial dependence was scored as zero points. A participant with a total Katz score below six during subsequent follow-up visits was categorised as having a disability. For participants who developed incident disability, the follow-up duration was defined as the interval between the time of first disability occurrence and the time of enrolment (completion of the second LA measurement). For participants who did not develop ADL disability, the censoring duration was defined as the interval between the time of the last valid follow-up and the time of enrolment.

### Covariates

Based on previous studies [10–12], we sequentially adjusted our multivariate analysis for the following covariates: age, sex, residence (rural or urban), ethnicity (Han or other), living alone, illiteracy, marital status, body mass index (BMI), current smoking, and alcohol consumption. Cognitive function was assessed using the Mini-Mental State Examination questionnaire, with cognitive impairment defined as a score of <18 for illiterate individuals or <24 for literate individuals [13]. Information on the history of chronic diseases, including hypertension, heart disease, cerebrovascular disease, diabetes, and respiratory diseases, was collected using a standard questionnaire.

### Statistical analysis

We used means and standard deviations to describe continuous data and frequencies with percentages to describe categorical variables. Characteristics between different LA groups were compared using χ^2^ tests or analysis of variance. We used proportional hazards models to analyse the relationship between changes in LA and the risk of disability, with the Cox proportional hazards assumption supported by Schoenfeld residual plots. Here, model 1 was adjusted for age and gender, model 2 was additionally adjusted for residence, ethnicity, living arrangement, illiteracy, marital status, smoking, alcohol consumption, and BMI, and model 3 was yet further adjusted for cognitive impairment, hypertension, diabetes, heart disease, cerebrovascular disease, and respiratory diseases. We used the sustained low group as the reference to calculate the hazard ratios (HRs) and 95% confidence intervals (CIs) in each model.

We also performed stratified analyses by sex (male, female) and age (<80 years, ≥80 years), and several sensitivity analyses to test the stability of the main results by excluding 378 participants aged >100 years, excluding participants who had heart disease (n = 870) or cerebrovascular disease (n = 394) at baseline, and additionally adjusting for recruitment time in model 3. We also employed inverse probability of treatment weighting (IPTW) as an alternative statistical strategy to control for potential confounders.

We performed all statistical analyses were performed using *R*, version 4.2.0 (R Foundation for Statistical Computing, Vienna, Austria), with a two-tailed *P*-value <0.05 denoting statistical significance.

## RESULTS

### Demographic characteristics

**Figure 1:**
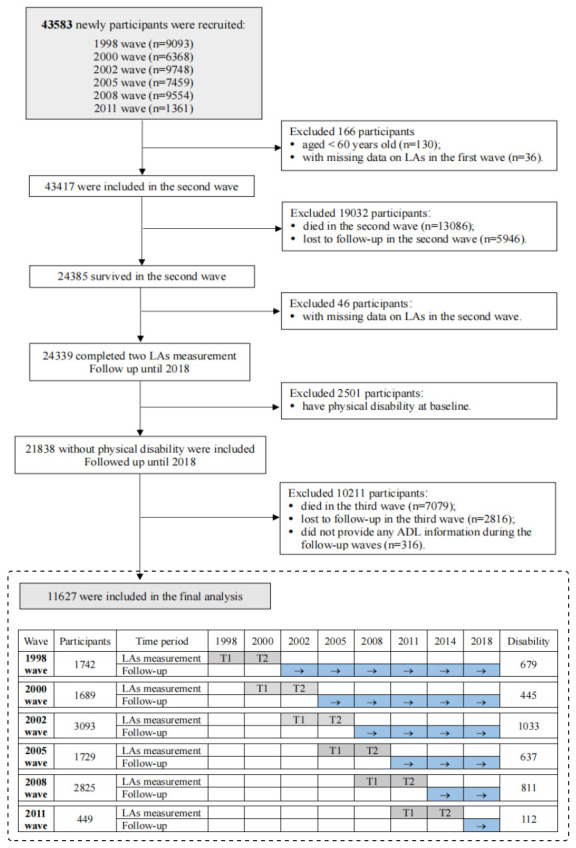
Flowchart of participant recruitment and follow-up interviews.

We first included 43583 individuals from the initial CLHLS dataset. Then, we excluded 130 participants who were <60 years old and 36 who were missing LA data in the first wave. In the second wave, 13 086 individuals died and 5946 were lost to follow-up, leaving 24385 observations. After excluding 46 individuals lacking LA data in the second wave and 2501 who had reported disabilities at baseline, 21838 participants remained in the follow-up. In the first follow-up wave, 7079 individuals died, 2816 were lost to follow-up and 316 failed to provide any ADL information. We finally included 11627 individuals in the final analysis (**Figure 1**) with a mean age of 79.46 years (standard deviation = 0.36), and with 52.03% being female. Most of the participants were of Han ethnicity (93.15%). Furthermore, 1745 individuals (15.01%) lived alone, and 1078 (9.27%) had cognitive impairment. Based on changes in LA participation, we categorised 2931 individuals in the sustained low LA group, 1729 in the LA increase group, 1802 in the LA decrease group, and 5165 in the sustained high LA group. We note statistically significant differences in the primary demographic characteristics and health status of the participants across changes in LA groups (**Table 1**).

**Table 1:** Demographic characteristics and disability rate of 11627 older adults across changes in LAs*

	Overall	Sustained low	LA increase	LA increase	Sustained high	F/ꭓ^2^	*P*-value
**Participants**	11627	2931	1729	1802	5165		
**Disability rate**	3717 (31.97)	1109 (37.84)	691 (39.97)	527 (29.25)	1390 (26.91)	162.91	<0.001
**Age in years, x̄ (SD)**	79.46 (10.36)	86.03 (9.35)	78.92 (9.87)	80.65 (9.46)	75.51 (9.36)	1943.79	<0.001†
**Female**	6050 (52.03)	2045 (69.77)	965 (55.81)	940 (52.16)	2100 (40.66)	659.97	<0.001
**Han ethnic**	10830 (93.15)	2671 (91.13)	1608 (93.00)	1700 (94.34)	4851 (93.92)	26.54	<0.001
**Rural**	6122 (52.65)	1438 (49.06)	1104 (63.85)	956 (53.05)	2624 (50.80)	110.78	<0.001
**Live alone**	1745 (15.01)	597 (20.37)	308 (17.81)	281 (15.59)	559 (10.82)	148.49	<0.001
**<1 y of schooling**	6392 (54.98)	2311 (78.85)	1082 (62.58)	1116 (61.93)	1883 (36.46)	1521.18	<0.001
**Current married**	5077 (43.67)	647 (22.07)	742 (42.91)	672 (37.29)	3016 (58.39)	1080.62	<0.001
**BMI in kg/m^2^, x̄ (SD)**	20.47 (3.92)	19.55 (3.81)	20.11 (3.75)	20.32 (3.85)	21.17 (3.93)	116.34	<0.001†
**Smoking**	2735 (23.52)	441 (15.05)	396 (22.90)	419 (23.25)	1479 (28.64)	201.48	<0.001
**Alcohol consumption**	2783 (23.94)	527 (17.98)	405 (23.42)	395 (21.92)	1456 (28.19)	114.68	<0.001
**Cognitive impairment**	1078 (9.27)	489 (16.68)	149 (8.62)	173 (9.60)	267 (5.17)	280.66	<0.001
**Hypertension**	1999 (17.19)	497 (16.96)	321 (18.57)	274 (15.21)	907 (17.56)	8.01	0.046
**Diabetes**	215 (1.85)	26 (0.89)	30 (1.74)	24 (1.33)	135 (2.61)	36.31	<0.001
**Heart disease**	870 (7.48)	155 (5.29)	117 (6.77)	118 (6.55)	480 (9.29)	49.07	<0.001
**Cerebrovascular disease**	394 (3.39)	76 (2.59)	58 (3.35)	56 (3.11)	204 (3.95)	11.33	0.013
**Respiratory disease**	1153 (9.92)	278 (9.48)	166 (9.60)	159 (8.82)	550 (10.65)	6.34	0.096

BMI – body mass index, LAs – leisure activities, SD – standard deviation, x̄ – mean

*Presented as n (%) unless specified otherwise.

†These *P*-values were generated using analysis of variance; the remaining ones were obtained via χ^2^ tests.

### Associations of changes in overall LAs with disability

A total of 3717 participants developed ADL disability during 51991.85 person-years of follow-up. The sustained low group had the highest disability incidence (142.04 per 1000 person-years), while the sustained high group had the lowest (46.71 per 1000 person-years). There were significant differences (*P* < 0.001) in the incidence of disability among the older population between four LA change patterns (**Table 1**). After adjusting for all covariates in the total sample, the adjusted hazard ratio (aHR) of disability for the sustained high group was 0.62 (95% CI = 0.57–0.69) with the sustained low group as the reference. An increase in LA was associated with a lower risk of disability (aHR = 0.66; 95% CI = 0.59–0.74). In subgroups stratified by sex and age, both the sustained high group and the LA increase group had a lower risk of disability compared to the sustained low group (**Table 2**). To observe the impact of changes in LAs on both short-term and long-term risks of disability, we further analysed the risk of disability over 3, 5, 10, and 15 years for all participants. We found that the effects of maintaining high participation and increasing participation in LAs on reducing the risk of disability were not influenced by the duration of follow-up (**Table 3**). We separately examined the associations between changes in physical LAs and cognitive LAs and the risk of disability. Similarly, sustained high physical LAs and sustained high cognitive LAs were associated with a 34% (aHR = 0.66; 95% CI = 0.60–0.73) and 27% (aHR = 0.73; 95% CI = 0.66–0.81) lower risk of disability, respectively. Increased participation in physical LAs and cognitive LAs was also associated with a lower risk of disability, with aHRs of 0.78 (95% CI = 0.71–0.86) and 0.85 (95% CI = 0.74–0.96), respectively (Table S1 in the **Online Supplementary Document**).

**Table 2:** Associations of changes in overall LAs with disability*

				Model 1		Model 2		Model 3	
	**Participant**	**Disability**	**Person years**	**HR (95% CI)**	***P*-value**	**HR (95% CI)**	***P*-value**	**HR (95% CI)**	***P*-value**
**Total sample**									
Sustained low	2931	1109	8916.06	ref		ref		ref	
Sustained high	5165	1390	27793.67	0.61 (0.56–0.66)	<0.001	0.60 (0.54–0.65)	<0.001	0.62 (0.57–0.69)	<0.001
LA increase	1729	691	7744.34	0.66 (0.59–0.73)	<0.001	0.63 (0.57–0.71)	<0.001	0.66 (0.59–0.74)	<0.001
LA decrease	1802	527	7537.79	0.98 (0.89–1.08)	0.723	0.98 (0.88–1.08)	0.652	1.01 (0.91–1.11)	0.885
**Men**									
Sustained low	886	284	2577.93	ref		ref		ref	
Sustained high	3065	762	15937.42	0.61 (0.53–0.70)	<0.001	0.59 (0.51–0.69)	<0.001	0.61 (0.52–0.71)	<0.001
LA increase	764	290	3296.20	0.68 (0.57–0.81)	<0.001	0.65 (0.54–0.77)	<0.001	0.66 (0.56–0.79)	<0.001
LA decrease	862	225	3496.12	1.08 (0.92–1.28)	0.336	1.08 (0.91–1.28)	0.359	1.07 (0.91–1.27)	0.386
**Women**									
Sustained low	2045	825	6338.13	ref		ref		ref	
Sustained high	2100	628	11856.24	0.59 (0.53–0.67)	<0.001	0.60 (0.54–0.67)	<0.001	0.64 (0.57–0.73)	<0.001
LA increase	965	401	4448.14	0.67 (0.58–0.78)	<0.001	0.64 (0.56–0.73)	<0.001	0.68 (0.59–0.78)	<0.001
LA decrease	940	302	4041.67	1.06 (0.93–1.19)	0.393	0.94 (0.83–1.06)	0.313	0.98 (0.86–1.11)	0.742
**<80 years**									
Sustained low	487	173	2981.41	ref		ref		ref	
Sustained high	3028	769	20857.54	0.62 (0.52–0.73)	<0.001	0.63 (0.53–0.74)	<0.001	0.64 (0.54–0.77)	<0.001
LA increase	805	246	5045.49	0.75 (0.61–0.92)	0.007	0.74 (0.60–0.92)	0.006	0.73 (0.58–0.91)	0.005
LA decrease	608	170	3949.16	0.84 (0.62–1.06)	0.185	0.87 (0.72–1.07)	0.193	0.87 (0.71–1.07)	0.184
**≥80 years**									
Sustained low	2444	936	5934.64	ref		ref		ref	
Sustained high	2137	621	6936.13	0.60 (0.54–0.67)	<0.001	0.57 (0.51–0.64)	<0.001	0.61 (0.54–0.68)	<0.001
LA increase	924	445	2698.85	0.65 (0.57–0.74)	<0.001	0.61 (0.54–0.69)	<0.001	0.65 (0.57–0.74)	<0.001
LA decrease	1194	357	3588.63	1.08 (0.97–1.22)	0.143	1.06 (0.94–1.19)	0.297	1.12 (0.98–1.25)	0.075

CI – confidence interval, HR – hazard ratio, LA – leisure activity, ref – reference

*Model 1 adjusted for age, sex (not in sex-subgroup analyses). Model 2 adjusted as model 1 and additionally for ethnicity, residence, living alone, illiterate, marital status, smoking, alcohol consumption, and BMI. Model 3 adjusted as model 2 and additionally for cognitive impairment, hypertension, diabetes, heart disease, cerebrovascular disease, and respiratory disease.

**Table 3:** Associations of changes in LAs with incident disability, stratified by follow-up periods*

	Participants	Disability	HR (95% CI)	*P*-value
**Three-year risk of disability**				
Sustained low	2931	661	ref	
Sustained high	5165	422	0.43 (0.37–0.49)	<0.001
LA increase	1729	359	0.56 (0.47–0.64)	<0.001
LA decrease	1802	226	1.07 (0.93–1.22)	0.319
**Five-year risk of disability**				
Sustained low	2931	914	ref	
Sustained high	5165	715	0.47 (0.42–0.52)	<0.001
LA increase	1729	506	0.59 (0.52–0.67)	<0.001
LA decrease	1802	355	1.02 (0.91–1.14)	0.751
**Ten-year risk of disability**				
Sustained low	2931	1078	ref	
Sustained high	5165	1238	0.56 (0.51–0.62)	<0.001
LA increase	1729	661	0.63 (0.56–0.69)	<0.001
LA decrease	1802	497	0.99 (0.90–1.10)	0.898
**Fifteen-year risk of disability**				
Sustained low	2931	1108	ref	
Sustained high	5165	1384	0.57 (0.52–0.63)	<0.001
LA increase	1729	689	0.63 (0.57–0.70)	<0.001
LA decrease	1802	526	0.98 (0.89–1.08)	0.717

CI – confidence interval, HR – hazard ratio, LA – leisure activity, ref – reference

*All analyses were adjusted for age, sex, ethnicity, residence, living arrangement, education, marital status, smoking, alcohol consumption, BMI, cognitive function, hypertension, diabetes, heart disease, cerebrovascular disease, and respiratory disease.

### Associations of changes in specific LAs with disability

**Figure 2:**
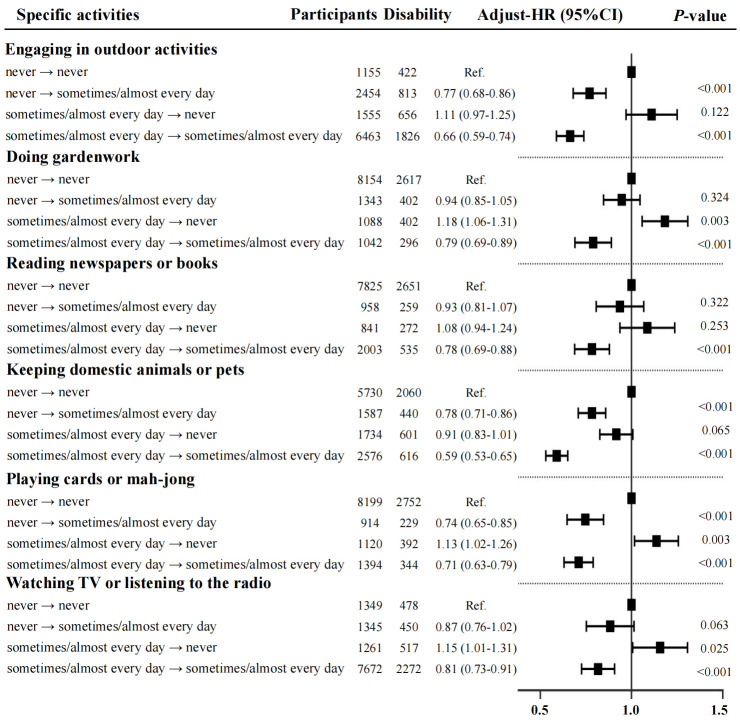
Associations of change in specific types of leisure activity with risk of disability. All analyses were adjusted for age, sex, education level, marital status, ethnicity, residence, living arrangements, BMI, smoking, drinking, cognitive function, hypertension, diabetes, cerebrovascular diseases, and respiratory diseases. CI – confidence interval, HR – hazard ratio.

Among all types of LAs, the group of sustained high participation (group 4) demonstrated the lowest risk of disability. Additionally, increased participation in outdoor activities (aHR = 0.77; 95% CI = 0.68–0.86), keeping domestic animals or pets (aHR = 0.78; 95% CI = 0.71–0.86), and playing cards or mahjong (aHR = 0.74; 95% CI = 0.65–0.85) were factors significantly associated with a lower risk of disability (*P* < 0.05) (**Figure 2**). We further conducted subgroup analyses by rural and urban residence to explore regional differences in the effects of specific LAs on disability. Gardening and reading newspapers or books showed significant heterogeneity between urban and rural areas (*P*-value for interaction <0.05), while the other four activities showed no significant residence-based differences (Table S2 in the **Online Supplementary Document****)**.

### Sensitivity analyses

The results of the sensitivity analyses did not substantially differ from the main findings (Figure S1 and Tables S1–8 in the **Online Supplementary Document**). Exclusion of participants aged >100 years, those with cerebrovascular disease, or those with heart disease did not substantially alter the associations between changes in LAs and disability. The overall conclusions also remained robust when additionally adjusting for recruitment time (in years). After applying IPTW weighting, the baseline characteristics of all participants across the four LAs change groups showed no statistically significant differences. In the subsequent IPTW-based regression analysis, we noted that sustained high LAs and increased LAs were associated with a 33% and 27% lower risk of disability, respectively. Standardised mean differences for each covariate were markedly reduced after weighting (all <0.1).

## DISCUSSION

Based on a representative longitudinal cohort of 11627 older adults, we observed that maintaining high engagement in LAs is associated with a markedly lower risk of developing disabilities, and that increased involvement in specific types of LAs over time was likewise associated with a substantial reduction in the risk of ADL disability.

The six specific activities used to measure LAs in this study are culturally common for older adults in China, which reinforces the contextual relevance of our findings. We observed a negative association between LAs and the risk of ADL disability, consistent with prior studies that highlighted the protective role of LAs or similar behaviours against disability, frailty, and mortality across diverse populations [14–16]. However, compared to existing research predominantly conducted in Western contexts, our study provides data on the Chinese older population. It is also the first to explore the impact of dynamic changes in LAs on the risk of disability. The health benefits of overall LAs may stem from their multifaceted nature, as they often combine physical, intellectual, and social components, thereby contributing collectively to support physical fitness, cognitive resilience, and social well-being [7,15,17]. This multidimensional engagement fosters mobility, cognitive health, and emotional stability, which are essential for maintaining independence and reducing the risk of disability in later life.

Compared with individuals exhibiting consistently low participation in LAs, those with sustained high engagement or increases in LA experienced a 38% (HR = 0.62; 95% CI = 0.57–0.69) and 34% (HR = 0.66; 95% CI = 0.59–0.74) reduction in disability risk, respectively. These findings align with prior research showing significant associations between dynamic changes in LAs and both mortality risk and other adverse outcomes [18–20]. In addition to questionnaire-assessed LAs, prior studies have also found that wearable device-measured physical activity is associated with a lower risk of functional disability among older adults [21,22]. Here, we found that the prevalence of cognitive impairment was lower in older individuals in the sustained high group and increasing LA group compared to those in the sustained low and decreasing LA groups. Our multivariable Cox regression analyses showed that sequential adjustments for lifestyles, cognitive function, and various chronic conditions attenuated the HRs, suggesting that these factors may partially explain the relationship between dynamic LA changes and disability risk. The bi-directional relationship between LA participation and cognitive function may help us understand the neurodegenerative mechanisms underlying the impact of dynamic changes in LAs on disability. Supporting this interpretation, several studies have demonstrated that shifts in LAs correlate with improved cognitive function, enhanced physical performance, and superior overall health outcomes [17,23]. Psychologically, maintained or increased LA engagement appears to enhance mood, emotional well-being, and a sense of purpose – all factors associated with reduced risks of ADL disability and cognitive decline [24,25]. Biologically, increased participation in LAs stimulates neural activity, modulates stress-related hormones, and improves cardiometabolic health – mechanisms linked to increased longevity and delayed functional impairment [26,27]. Socially, increased engagement in LAs fosters interactions and support networks that have been empirically linked to better physical and mental health outcomes, including a slower progression of ADL disability [17]. Behaviourally, LAs promote active lifestyles, encourage adherence to healthy behaviours, and reduce engagement in detrimental activities, collectively mitigating the risk of chronic disease and falls [28,29]. Taken together, these results underscore the importance of maintaining or increasing participation in LAs as a protective strategy against disability in older adults.

Our findings further suggested that the positive effects of increased participation in LAs have equal benefits for males and females, which differs from previous findings [30]. Besides, older individuals aged 80 years or older who consistently maintained high levels of LAs participation or increased their participation over time experienced a lower risk of mortality [8], suggesting that increased and sustained involvement in LAs might enhance their lifespan [31,32]. Given that 57.62% of our participants were over 80 years old, our findings suggest that engaging in LAs can yield benefits even in later stages of life. Furthermore, compared to the increasing participation group, those with sustained high participation had significantly lower risk of developing disabilities, reinforcing the notion that earlier initiation of LAs is associated with a greater likelihood of maintaining health in old age. Even in the final stages of life, increased engagement in LAs could play a crucial role in preventing disability while promoting independence and enhancing quality of life.

Our study provides evidence on which specific LAs may reduce the risk of disability among older adults. Increased outdoor recreation or gardening participation, for example, could improve mobility among this population. Such activities were previously shown to help the older individuals maintain flexibility and increase vitamin D exposure [33]. Outdoor activities in particular offer a low-impact form of exercise that can reduce the risk of chronic diseases and enhance overall fitness [34]. Gardening, meanwhile, requires adequate open space for plants, which could be inaccessible to older adults in urban areas, who could consider container gardening or community gardens as viable alternatives, fostering not only physical activity, but also interpersonal interactions among their peers. Similarly, public health practitioners should carefully differentiate between recreational gardening and farming when recommending outdoor activities or gardening to older adults in rural areas to avoid potential misunderstandings. Previous studies indicated that increased exposure to farming or construction work might contribute to the occurrence of fractures in older males [35], while among females, farmers and retired individuals had a higher incidence of traumatic spinal fractures [36]. Thus, we recommend that future public health interventions focus on creating open activity areas or community gardens in urban and rural regions and providing tailored guidance to older adults.

Keeping pets or domestic animals can help alleviate feelings of loneliness and being undervalued, which are commonly reported by retired individuals, as the companionship, emotional support, and sense of purpose derived from caring for an animal have been shown to combat these negative perceptions [10,11,37,38]. Having a dog, for example, would encourage outdoor walking and increase opportunities for social interactions [39]. However, it is essential to consider the physical and financial capabilities of older individuals when recommending pet or domestic animal ownership. In this sense, low-maintenance pets or domestic animals might be more suitable for older persons who face such limitations [40]. We also note that the loss of a pet could contribute to a significant emotional trauma in older adults. To mitigate this, it is crucial to design interventions that improve coping skills and increase the availability of community-based mental health support for this population [41,42].

Our study highlights the importance of culturally specific LAs for Chinese older adults, particularly the need for outdoor activities and cognitively stimulating games to support the goals of healthy ageing. As urbanisation accelerates in China, the development of community parks and green spaces will become crucial for enhancing residents' quality of life. Unlike expensive fitness facilities or organised sports, community park-based outdoor activities provide a low-cost and inclusive option that is accessible to older adults of all socioeconomic backgrounds. Community parks also serve as social hubs for interaction among residents that could help mitigate the social isolation often caused by rapid urbanisation, with studies showing that proximity to green spaces is associated with reduced chronic conditions and improved mental health[43,44].

Playing cards or mahjong are deeply embedded in Chinese social traditions and differ from Western cognitive games in their accessibility, group-oriented nature, and integration into daily life. The theoretical foundation behind these activities aligns with the cognitive reserve hypothesis, which suggests that engaging in mentally stimulating tasks can enhance brain plasticity and delay cognitive decline [45,46]. Unlike many Western intelligence games that require formal education or structured learning, playing cards or mahjong allows older adults to engage in complex cognitive tasks, such as recognising symbols, numbers, and patterns, without the prerequisite of literacy or formal schooling. This makes them particularly beneficial for individuals with limited reading skills, promoting self-efficacy and long-term cognitive and emotional well-being [47]. In addition to cognitive benefits, playing cards or mahjong contributes to sensorimotor function by improving hand-eye coordination and fine motor skills [48], which are critical for maintaining functional independence in later life. These games also serve as a key form of social infrastructure, counteracting the effects of urbanisation and family separation.

Several limitations should be noted. First, although diverse lifestyles and chronic diseases were considered in the multivariate analysis, the influence of unmeasured confounders such as social support, neighbourhood environmental factors, and early-life physical activity cannot be ruled out. Second, our participants were recruited from communities in China only, limiting the generalisability of our findings to other regions and ethnic groups. Third, LAs were measured only twice, preventing us from capturing detailed longitudinal trajectories and temporal changes of LAs in older adults. Fourth, semiquantitative questionnaires cannot accurately capture older adults’ activity levels, and device-based measurements such as accelerometry should therefore be employed in future studies. Fifth, since the CLHLS cohort focusses on longevity, the mean age of our participants is higher than that of the general older population in China. Therefore, loss to follow-up and deaths during sample exclusion may have introduced some selection bias. Finally, we only included disability in ADL as an outcome in this study; the impact of changes in LAs on different severities and phenotypes of disability should be explored in future research.

## CONCLUSION

Our findings indicate that maintaining high participation in LAs is associated with a lower risk of ADL disability among older adults in China. Specific activities appear to provide multifaceted benefits by engaging different aspects of well-being simultaneously. For example, outdoor activities and pet keeping offer physical exercise and mobility training, while playing cards or mahjong provides cognitive stimulation and social engagement. These results emphasise the need for culturally tailored interventions to encourage healthy ageing, enhance functional independence, and improve the quality of life among older populations.

## Additional material


Online Supplementary Document
