# Supplementary material for: Impact of changes in leisure activities on incident disability among older adults: a nationwide cohort study
Source: J Glob Health. 2025 Oct 31;15:04289. doi: 10.7189/jogh.15.04289 (PMC12577667; doi:10.7189/jogh.15.04289)
Supplement: Online Supplementary Document [file jogh-15-04289-s001.pdf]

Supplement to: Zhang C, Liu J, Kang Y, Gao Q, Zhang J, Zhang Y, Zhao Y, Zeng P, Shen J. Impact of changes in leisure activities on incident disability among older adults: a nationwide cohort study. J Glob Health. 2025;15:04289.

Supplementary materials

Table S1. Associations of changes in physical and cognitive leisure activities with disability

Groups	Participant	Disability	Person years	Fully adjusted model	
				HR (95% CI)	*P*-value
**Physical LAs**					
Sustained low	5899	2007	21116.29	Ref.	
Sustained high	1955	504	11683.04	0.66 (0.60-0.73)	<0.001
LAs increase	1760	514	8781.86	0.78 (0.71-0.86)	<0.001
LAs decrease	2013	692	10410.65	0.96 (0.88-1.05)	0.417
**Cognitive LAs**					
Sustained low	7088	2410	29121.16	Ref.	
Sustained high	2354	619	12286.05	0.73 (0.66-0.81)	<0.001
LAs increase	1061	294	5265.29	0.85 (0.74-0.96)	0.009
LAs decrease	1124	394	5319.37	1.13 (1.01-1.27)	0.024

Note: Adjusted for age, sex, ethnicity, residence, living arrangement, education, marital status, smoking, alcohol consumption, BMI, cognitive function, hypertension, diabetes, heart disease, cerebrovascular disease, and respiratory disease.
LAs, leisure activities; HR, hazard ratios; CI, confidence interval.

Table S2. Associations of change in specific types of leisure activity with risk of disability across rural and urban residence

Changes in specific types of LAs	Rural			Urban			*P*-interaction
	Participants	Disability	Adj-HR (95%CI)	Participants	Disability	Adj-HR (95%CI)	
**Engaging in outdoor activities**							0.089
never → never	556	184	Ref.	599	238	Ref.	
never → sometimes/almost every day	1024	284	0.74(0.61-0.89)	1430	529	0.79(0.68-0.93)	
sometimes/almost every day → never	1009	390	1.08(0.91-1.29)	546	266	1.12(0.94-1.34)	
sometimes/almost every day → sometimes/almost every day	3533	976	0.71(0.60-0.83)	2930	850	0.61(0.52-0.71)	
**Doing gardenwork**							0.007
never → never	4622	1391	Ref.	3532	1226	Ref.	
never → sometimes/almost every day	701	212	1.13(0.97-1.31)	642	190	0.81(0.69-0.94)	
sometimes/almost every day → never	487	162	1.37(1.16-1.62)	601	240	1.04(0.91-1.21)	
sometimes/almost every day → sometimes/almost every day	312	69	0.81(0.63-1.03)	730	227	0.76(0.65-0.88)	
**Reading newspapers or books**							0.032
never → never	4538	1457	Ref.	3287	1194	Ref.	
never → sometimes/almost every day	555	146	1.01(0.84-1.21)	403	113	0.86(0.70-1.06)	
sometimes/almost every day → never	437	109	0.93(0.75-1.15)	404	163	1.24(1.03-1.49)	
sometimes/almost every day → sometimes/almost every day	592	122	0.92(0.71-1.18)	1411	413	0.75(0.65-1.88)	
**Keeping domestic animals or pets**							0.301
never → never	2195	786	Ref.	3535	1274	Ref.	
never → sometimes/almost every day	969	245	0.69(0.61-0.81)	618	195	0.87(0.75-1.02)	
sometimes/almost every day → never	1105	366	0.88(0.78-1.01)	629	235	0.93(0.81-1.08)	
sometimes/almost every day → sometimes/almost every day	1853	437	0.56(0.49-0.64)	723	179	0.59(0.51-0.69)	
**Playing cards or mah-jong**							0.874
never → never	4459	1414	Ref.	3740	1338	Ref.	
never → sometimes/almost every day	483	1074	0.72(0.59-0.89)	431	125	0.81(0.66-0.96)	
sometimes/almost every day → never	556	171	1.13(0.96-1.33)	564	221	1.12(0.97-1.31)	
sometimes/almost every day → sometimes/almost every day	624	145	0.69(0.58-0.83)	770	199	0.72(0.62-0.84)	
**Watching TV or listening to the radio**							0.128
never → never	719	247	Ref.	630	231	Ref.	
never → sometimes/almost every day	790	238	0.82(0.69-0.99)	555	212	0.94(0.78-1.14)	
sometimes/almost every day → never	709	263	1.09(0.91-1.31)	552	254	1.24(1.03-1.49)	
sometimes/almost every day → sometimes/almost every day	3904	1086	0.84(0.72-0.97)	3768	1186	0.79(0.67-0.93)	

Note: Adjusted for age, sex, ethnicity, living arrangement, education, marital status, smoking, alcohol consumption, BMI, cognitive function, hypertension, diabetes, heart disease, cerebrovascular disease, and respiratory disease.

LAs, leisure activities; Adj-HR, adjusted hazard ratio; CI, confidence interval.

Table S3. Associations of change in total leisure activities with disability after excluding 378 participants aged >100 years

11294 participants were included

Groups	Participant	Disability	Person years	Fully adjusted model	
				HR (95% CI)	*P*-value
**Total sample**					
Sustained low	2697	1021	8564.59	Ref.	
Sustained high	5110	1375	27666.49	0.58 (0.53-0.64)	<0.001
LAs increase	1686	664	7676.40	0.64 (0.57-0.71)	<0.001
LAs decrease	1756	510	7457.45	0.98 (0.89-1.08)	0.738

Note: Adjusted for age, sex, ethnicity, residence, living arrangement, education, marital status, smoking, alcohol consumption, BMI, cognitive function, hypertension, diabetes, heart disease, cerebrovascular disease, and respiratory disease.
LAs, leisure activities; HR, hazard ratios; CI, confidence interval.

Table S4. Associations of change in total leisure activities with disability after excluding 870 participants with heart disease

10757 participants were included

Groups	Participant	Disability	Person years	Fully adjusted model	
				HR (95% CI)	*P*-value
**Total sample**					
Sustained low	2776	1042	8479.63	Ref.	
Sustained high	4685	1248	25437.67	0.57 (0.52-0.63)	<0.001
LAs increase	1612	643	7245.10	0.64 (0.57-0.72)	<0.001
LAs decrease	1684	495	7089.09	0.99 (0.94-1.10)	0.846

Note: Adjusted for age, sex, ethnicity, residence, living arrangement, education, marital status, smoking, alcohol consumption, BMI, cognitive function, hypertension, diabetes, heart disease, cerebrovascular disease, and respiratory disease.
LAs, leisure activities; HR, hazard ratios; CI, confidence interval.

Table S5. Associations of change in total leisure activities with disability after excluding 394 participants with cerebrovascular disease

11233 participants were included

Groups	Participant	Disability	Person years	Fully adjusted model	
				HR (95% CI)	*P*-value
**Total sample**					
Sustained low	2855	1073	8689.14	Ref.	
Sustained high	4961	1318	26868.31	0.58 (0.53-0.63)	<0.001
LAs increase	1671	663	7529.78	0.63 (0.57-0.71)	<0.001
LAs decrease	1746	506	7292.64	0.99 (0.89-1.09)	0.857

Note: Adjusted for age, sex, ethnicity, residence, living arrangement, education, marital status, smoking, alcohol consumption, BMI, cognitive function, hypertension, diabetes, heart disease, cerebrovascular disease, and respiratory disease.
LAs, leisure activities; HR, hazard ratios; CI, confidence interval.

Table S6. Associations of change in total leisure activities with disability after additionally adjusting for recruitment time

11627 participants were included

Groups	Participant	Disability	Person years	Fully adjusted model	
				HR (95% CI)	*P*-value
**Total sample**					
Sustained low	2931	1109	8916.06	Ref.	
Sustained high	5165	1390	27793.67	0.57 (0.52-0.63)	<0.001
LAs increase	1729	691	7744.34	0.62 (0.56-0.69)	<0.001
LAs decrease	1802	527	7537.79	0.97 (0.87-1.07)	0.531

Note: Adjusted for age, sex, ethnicity, residence, living arrangement, education, marital status, smoking, alcohol consumption, BMI, cognitive function, hypertension, diabetes, heart disease, cerebrovascular disease, respiratory disease, and recruitment time (in year).
LAs, leisure activities; HR, hazard ratios; CI, confidence interval.

Table S7. Baseline characteristics of 11627 older adults after using IPTW

Characteristics	Overall	Sustained low	LAs increase	LAs decrease	Sustained high	*P*-value	SMD
Participants, n	11627	2931	2936.66	2908.72	3092.27		
Age, year	79.46±10.36	86.03 (9.35)	85.84 (9.19)	85.99 (9.56)	87.23 (10.18)	0.068	0.072
Female, n (%)	6050 (52.03)	2045.0 (69.8)	2054.1 (69.9)	2003.0 (68.9)	2210.6 (71.5)	0.461	0.029
Han ethnic, n (%)	10830 (93.15)	2671.0 (91.1)	2661.9 (90.6)	2685.3 (92.3)	2796.5 (90.4)	0.513	0.036
Rural, n (%)	6122 (52.65)	1438(49.06)	1400.96(47.71)	1451.92(49.91)	1603.07(51.84)	0.271	0.044
Live alone, n (%)	1745 (15.01)	597.0 (20.4)	614.4 (20.9)	589.8 (20.3)	633.9 (20.5)	0.977	0.008
< 1 year schooling, n (%)	6392 (54.98)	2311.0 (78.8)	2319.5 (79.0)	2278.1 (78.3)	2450.1 (79.2)	0.915	0.012
Current married, n (%)	5077 (43.67)	647.0 (22.1)	638.8 (21.8)	677.9 (23.3)	621.9 (20.1)	0.124	0.040
Body mass index, kg/m^2	20.47±3.92	19.56 (3.81)	19.44 (3.62)	19.80 (3.89)	19.63 (3.53)	0.189	0.052
Smoking, n (%)	2735 (23.52)	441(15.04)	425.76(14.49)	461.52(15.86)	449.07(14.52)	0.659	0.022
Alcohol consumption, n (%)	2783 (23.94)	527(17.98)	532.76(18.14)	504.12(17.33)	543.57(17.58)	0.940	0.012
Cognitive impairment, n (%)	1078 (9.27)	489.0 (16.7)	483.3 (16.5)	489.2 (16.8)	572.0 (18.5)	0.719	0.027
Hypertension, n (%)	1999 (17.19)	497.0 (17.0)	499.5 (17.0)	477.3 (16.4)	554.4 (17.9)	0.793	0.021
Diabetes, n (%)	215 (1.85)	26.0 (0.9)	28.7 (1.0)	16.5 (0.6)	33.5 (1.1)	0.251	0.032
Heart disease, n (%)	870 (7.48)	155.0 (5.3)	152.0 (5.2)	164.4 (5.7)	167.9 (5.4)	0.944	0.012
Cerebrovascular disease, n (%)	394 (3.39)	76.0 (2.6)	80.4 (2.7)	88.8 (3.1)	65.6 (2.1)	0.394	0.031
Respiratory disease, n (%)	1153 (9.92)	278.0 (9.5)	273.8 (9.3)	301.3 (10.4)	257.2 (8.3)	0.368	0.036

Notes: LAs, leisure activities; IPTW, inverse probability of treatment weighting; SMD, standardized mean difference

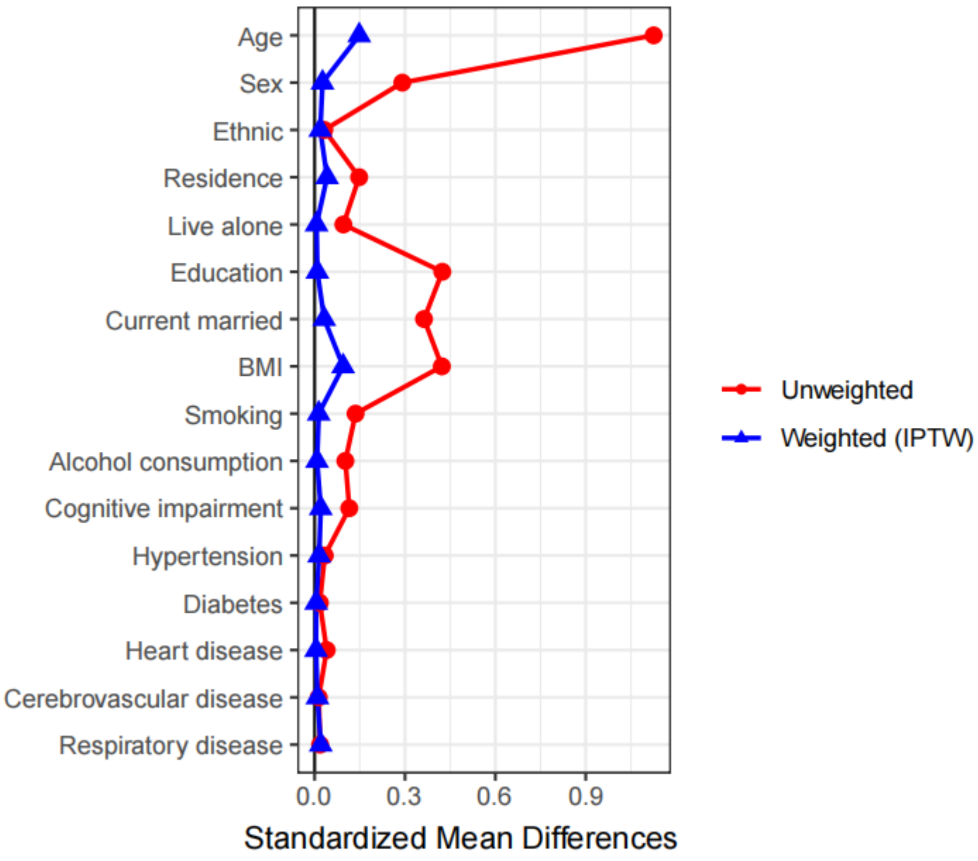

Figure S1. Comparison of standardized mean differences in baseline characteristics before and after inverse probability of treatment weighting

Table S8. Associations of changes in overall leisure activities with disability using inverse probability of treatment weighting

**Changes in LAs**	**Cox model using IPTW**			
	HR	95% CI Lower	95% CI Upper	*P*-value
Sustained low	Ref.			
Sustained high	0.67	0.61	0.72	<0.001
LAs increase	0.73	0.67	0.81	<0.001
LAs decrease	1.09	0.89	1.28	0.619

Note: LAs, leisure activities; IPTW, inverse probability of treatment weighting; HR, hazard ratios; CI, confidence interval

Table S9. Outline of JoGH's Guidelines for Reporting Analyses of Big Data Repositories Open to the Public (GRABDROP) items

JoGH guideline item	
1. Please list all papers published by each co-author in previous three years that were based on secondary analysis of a big data repository.	DOI: 10.1016/j.puhe.2025.105833<br>DOI: 10.7189/jogh.15.04119<br>DOI: 10.1093/gerona/glae301<br>DOI: 10.7189/jogh.14.04169<br>DOI: 10.1016/j.heliyon.2024.e29734<br>DOI: 10.1016/j.jad.2024.02.015<br>DOI: 10.1186/s12877-024-04706-x<br>DOI: 10.1016/j.puhe.2023.11.022<br>DOI: 10.1016/j.scitotenv.2023.169031<br>DOI: 10.1016/j.jad.2023.08.060
2. Please explain the key elements of your study design and the use of the available datasets that make your study an original scientific contribution.	This study utilized longitudinal data (1998-2018) from the Chinese Longitudinal Healthy Longevity Survey (CLHLS) to analyze the relationship between dynamic changes in leisure activities (LAs) and incident disability. To the best of our knowledge, the association of LA trajectories with disability risk had not been previously investigated in this cohort. Furthermore, we examined the impact of changes in specific activity types and assessed the consistency of these associations across different follow-up periods. These analyses collectively represent the original scientific contribution of our work to the field of healthy aging.
3. Please list all publications that addressed similar research questions in the same dataset and indicate where you cited them in your paper.	We acknowledge two previously published articles (Li, 2020 and Zhong, 2024; now cited as References 10 and 11) that explored the association between leisure activities (LAs) and disability using the CLHLS dataset. However, a key distinction is that those studies measured LAs at a single time point, whereas our analysis focuses specifically on changes in LAs over time, which had not been investigated for this specific outcome.<br>Furthermore, while our team recently published a study on the relationship between changes in LAs and all-cause mortality (doi: 10.7189/jogh.15.04119), it is crucial to note that the two studies have entirely different primary outcomes and inclusion/exclusion criteria, despite utilizing the same CLHLS dataset.
4. Please explain how you addressed multiple testing through an appropriately rigorous statistical threshold and indicate this in the methods section.	To ensure the robustness of our findings and address potential concerns regarding multiple testing, we employed a multi-pronged analytical strategy. The stability of our primary results was rigorously tested through stratification by key covariates (sex, residence, follow-up duration), multiple sensitivity analyses (e.g., excluding older participants or those with baseline cardiovascular diseases), and additional model adjustments. Moreover, the application of inverse probability of treatment weighting (IPTW), a distinct methodological approach, further validated our conclusions. The consistency of results across all these methods provides strong evidence that our findings are robust and not merely due to multiple comparisons.
5. Please declare to what extent have AI chatbots been used in developing your paper and to which parts of the paper did they contribute.	The authors utilized an AI-assisted tool exclusively for the purpose of language polishing and grammar checking in this manuscript. All intellectual content, including study design, data analysis, interpretation, and drafting of the original text, was solely performed by the human authors.
